# Exploration of a potent PI3 kinase/mTOR inhibitor as a novel anti-fibrotic agent in IPF

**DOI:** 10.1136/thoraxjnl-2015-207429

**Published:** 2016-04-21

**Authors:** Paul F Mercer, Hannah V Woodcock, Jessica D Eley, Manuela Platé, Michal G Sulikowski, Pascal F Durrenberger, Linda Franklin, Carmel B Nanthakumar, Yim Man, Federica Genovese, Robin J McAnulty, Shuying Yang, Toby M Maher, Andrew G Nicholson, Andy D Blanchard, Richard P Marshall, Pauline T Lukey, Rachel C Chambers

**Affiliations:** 1 Centre for Inflammation and Tissue Repair, UCL Respiratory, Rayne Institute, University College London, London, UK; 2 Department of Fibrosis DPU, Respiratory TA, GlaxoSmithKline, Stevenage, UK; 3 Nordic Bioscience, Herlev, Denmark; 4 NIHR Respiratory Biomedical Research Unit, Royal Brompton Hospital, London, UK

**Keywords:** Idiopathic pulmonary fibrosis

## Abstract

**Rationale:**

Idiopathic pulmonary fibrosis (IPF) is the most rapidly progressive and fatal of all fibrotic conditions with no curative therapies. Common pathomechanisms between IPF and cancer are increasingly recognised, including dysfunctional pan-PI3 kinase (PI3K) signalling as a driver of aberrant proliferative responses. GSK2126458 is a novel, potent, PI3K/mammalian target of rapamycin (mTOR) inhibitor which has recently completed phase I trials in the oncology setting. Our aim was to establish a scientific and dosing framework for PI3K inhibition with this agent in IPF at a clinically developable dose.

**Methods:**

We explored evidence for pathway signalling in IPF lung tissue and examined the potency of GSK2126458 in fibroblast functional assays and precision-cut IPF lung tissue. We further explored the potential of IPF patient-derived bronchoalveolar lavage (BAL) cells to serve as pharmacodynamic biosensors to monitor GSK2126458 target engagement within the lung.

**Results:**

We provide evidence for PI3K pathway activation in fibrotic foci, the cardinal lesions in IPF. GSK2126458 inhibited PI3K signalling and functional responses in IPF-derived lung fibroblasts, inhibiting Akt phosphorylation in IPF lung tissue and BAL derived cells with comparable potency. Integration of these data with GSK2126458 pharmacokinetic data from clinical trials in cancer enabled modelling of an optimal dosing regimen for patients with IPF.

**Conclusions:**

Our data define PI3K as a promising therapeutic target in IPF and provide a scientific and dosing framework for progressing GSK2126458 to clinical testing in this disease setting. A proof-of-mechanism trial of this agent is currently underway.

**Trial registration number:**

NCT01725139, pre-clinical.

**Key messages**

**What is the key question?**

- Is there a scientific rationale for the use of a clinically advanced pan-PI3 kinase (PI3K)/mammalian target of rapamycin (mTOR) inhibitor in idiopathic pulmonary fibrosis (IPF)?

**What is the bottom line?**

- We provide a comprehensive scientific and dosing framework for targeting PI3 kinase signalling in IPF with the pan-PI3 kinase/mTOR inhibitor, GSK2126458, based on the exploration of novel functional endpoints in patient-derived cells and precision cut lung slices.

**Why read on?**

- This study provides key data to support the progression of this agent to a proof-of-mechanism trial in patients with IPF and further proposes a roadmap for the repositioning of existing oncology agents as novel therapeutic agents in the IPF setting with minimal requirement for the additional use of experimental animals.

## Introduction

Idiopathic pulmonary fibrosis (IPF) represents one of the most rapidly progressive fibrotic conditions with a median survival of 3.5 years from diagnosis,^1^ worse than many cancers. The last decade has seen an exponential rise in clinical trial activity in IPF with agents which exert pleiotropic effects, such as pirfenidone (Esbriet) and nintedanib (Ofev) recently granted approval as the first agents to slow disease progression.^2^ ^3^ However, there still remains an urgent need to develop therapeutic strategies which halt rather than slow disease progression.

Fibrotic foci, the sentinel lesions in IPF, comprise fibroblasts and α smooth muscle actin positive (αSMA+) myofibroblasts embedded in a type I collagen-rich matrix. These lesions are thought to represent the leading edge of the relentless fibrotic response and arise as a result of a highly abnormal wound healing response characterised by dysregulated epithelial/mesenchymal crosstalk and a complex network of proliferation and differentiation signals received from a remodelled and homeostatically degenerate microenvironment^4–7^ (reviewed in^4^). Parallels between IPF and cancer are increasingly recognised, with active signalling pathways, transcriptomic and miRNA profiles and epigenetic signatures showing a considerable degree of overlap with lung cancer.^8–11^ Moreover, increased 18F-fluorodeoxyglucose (FDG) uptake, a marker of aerobic glycolysis in cancer cells, was recently reported in the lungs of patients with IPF.^12^ Together these observations support the notion that a number of pharmacological agents developed for oncology might be explored as potential therapeutic agents in the setting of IPF.

Class 1 PI3K form an important oncogenic signalling node, integrating signals from a variety of inputs including tyrosine kinase receptors, G-protein coupled receptors and activated Ras,^13^ to mediate a variety of cellular processes, including cell cycle progression, growth and proliferation, metabolic and synthetic pathways in addition to inflammatory responses. There is evidence that class 1 PI3K isoforms, most notably p110γ, exhibit increased expression in IPF tissue and fibroblast lines,^14^ with signalling activated downstream of several key profibrotic growth factors implicated in IPF, including platelet-derived growth factor and transforming growth factor (TGF)-β_1._^15^ ^16^ Evidence suggests that dysregulated PI3K signalling due to low activity of the tumour suppressor, phosphatase and tensin homologue (PTEN) may contribute to the profibrotic phenotype of IPF myofibroblasts,^17^ ^18^ and moreover that inhibition of class 1 PI3K has anti-fibroproliferative effects *in vitro* and in tumour necrosis factor α induced fibrosis *in vivo*.^19^ ^20^

GSK2126458 is a potent, highly selective pyridilsulfonamide inhibitor of class 1 isozymes of PI3K in addition to mammalian target of rapamycin (mTOR).^21^ Originally developed in the oncology setting, GSK2126458 is currently (at the time of writing) being evaluated in a phase I open-label dose escalation trial in subjects with refractory solid tumours or lymphoma (https://clinicaltrials.gov/ct2/show/NCT00972686).

The aim of the present study was to evaluate the scientific and dose rationale for re-positioning GSK2126458 as a potential novel therapeutic agent for IPF. We explore evidence for PI3K pro-fibrotic signalling in IPF lung tissue in combination with performing *in vitro* pharmacological assessment of GSK2126458 and related development compounds in lung IPF and non-IPF control fibroblasts. We also assess inhibitor effects using novel functional endpoints in precision-cut IPF tissue slices, and further evaluate pAkt levels in IPF bronchoalveolar (BAL) cells as a potential pharmacodynamic (PD) biomarker to monitor target engagement in future clinical studies. Integration of these data with pharmacokinetic (PK) data for GSK2126458 from oncology trials enabled modelling of an optimal dosing regimen for IPF and the progression of GSK2126458 to an IPF proof-of-mechanism (PoM) study (https://clinicaltrials.gov/ct2/show/NCT01725139). As well as providing a rationale for progressing a novel therapeutic agent in IPF, these studies further provide a new paradigm for the future re-positioning of existing drugs as novel anti-fibrotic agents.

## Methods

### Patient material

All human samples were obtained with informed signed consent and with research ethics committee approval (10/H0504/9, 10/H0720/12 and 12/EM/0058). Patients with IPF were diagnosed in accordance with current international guidelines.^22^ Tissue for lung slice experiments was obtained from Asterand Europe (Royston, UK) in compliance with the UK Human Tissue Act 2004.

### Primary fibroblast and bronchial epithelial cell culture

Primary human lung fibroblasts (LFs) were grown from explant cultures of IPF (IPF-LF) or non-IPF control (C-LF) lung tissue, and cultured as previously described.^23^ Primary human bronchial epithelial cells (HBECs) were isolated from regions of normal airway from IPF or non-IPF control lung tissue as previously described.^24^ See online supplementary material.

10.1136/thoraxjnl-2015-207429.supp1 Supplementary data

### Immunohistochemistry

Immunostaining for pAkt^S473^, pAkt^T308^, αSMA and CD68 was conducted on 4 μm formalin-fixed paraffin-embedded serial sections of human lung biopsy material from n=12 patients with IPF, using the avidin–biotinylated HRP enzyme complex method (Vector Laboratories). Confocal dual-immunofluorescence microscopy for these markers was also performed on representative fibrotic foci. See online supplementary material.

### Akt phosphorylation and collagen synthesis in precision-cut lung slices

Precision cut slices of IPF lung, 8 mm, were cultured for 24 h in DMEM supplemented with 0.4% FCS (5% CO_2_/100% humidity) prior to incubation with the PI3K inhibitors Compound 1 or GSK2126458. The effect of short-term exposure (2 h) to Compound 1 on Akt phosphorylation was assessed by Milliplex Map (Millipore) bead assay of lung slice homogenates prepared in ice cold PhosphoSafe buffer (Millipore). In separate experiments, the effect of GSK2126458 on levels of the collagen formation marker P1NP in tissue supernatant was assessed by proprietary competitive ELISA (Nordic Bioscience) as previously described.^25^ Slices were incubated with inhibitor for up to 5 days prior to harvest. See online supplementary material.

### Fibroblast and BALF fluid cell phospho-proteins

The effect of GSK2126458 on FCS-induced Akt phosphorylation in C-LFs (n=2) and IPF-LFs (n=5) was assessed by Meso Scale Discovery (MSD) system. Phosphorylation events (pAkt and pSMAD2) in C-LFs were also assessed by Western blot. Densitometric analysis of Western blots was performed using ImageQuant TL v8.1 software (GE Healthcare). The intensity of bands in phospho-protein blots was divided by the intensity of the total protein following stripping and re-probing. Values were expressed as a ratio of the positive (stimulated) control. The effect of GSK2126458 on bronchoalveolar lavage fluid (BALF) cell pellet obtained from patients with IPF (n=6) undergoing routine diagnostic BAL was also assessed by MSD. See online supplementary material.

### Fibroblast proliferation

The effect of GSK2126458 on FCS-induced proliferation in control (n=2) and IPF-derived primary human LFs (n=5) was assessed by MTS assay. Fibroblast proliferation was also assessed by 5-ethynyl-2′deoxyuridine (EdU) incorporation into DNA of primary human LFs (n=5 control, n=2 IPF). Fibroblast and HBEC apoptosis was assessed by caspase 3,7 activation. See online supplementary material.

### Determination of collagen 1 gene expression and Pro-collagen synthesis

Col1A1 gene expression in confluent fibroblast monolayers following TGFβ stimulation was assessed by RT-PCR following TRIzol extraction as per the manufacturer's instructions (Thermo Fisher). RNA was DNAse treated using a DNAfree kit (Ambion) and real-time RT-PCR conducted using the MESA GREEN qPCR MasterMix Plus for SYBR Assay (Eurogentec). Detailed methods are provided in the online supplementary material. Fibroblast pro-collagen production was assessed by high-performance liquid chromatography (HPLC) quantitation of hydroxyproline in supernatants from confluent fibroblast monolayers, as previously described.^26^ Collagen biosynthesis and deposition was also assessed by molecular crowding assay modified from previously described methods using the IN Cell Analyzer 6000 high content imaging system (GE Healthcare Life Sciences).^27^ See online supplementary material.

## Results

### Active PI3K signalling within IPF fibrotic foci

**Figure 1:**
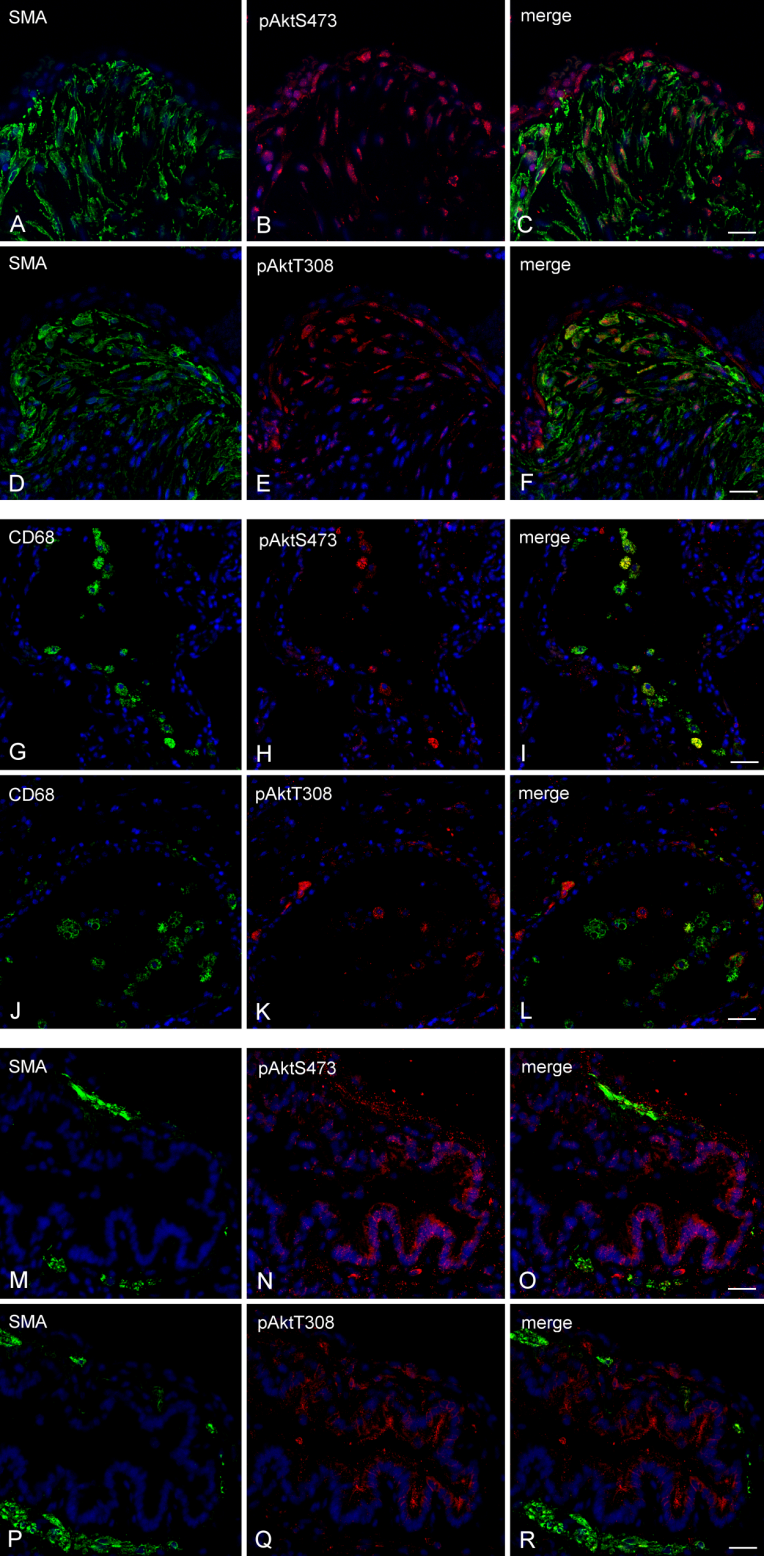
Akt phosphorylation co-localised within fibrotic foci, macrophages and bronchial epithelium in the idiopathic pulmonary fibrosis (IPF) lung. Immunofluorescence for α smooth muscle actin (αSMA, green, A and D), Akt phosphorylated at serine 473 (pAkt^S473^, red, B) and threonine 308 (pAkt^T308^, red, E) is shown co-localised (C and F) within fibrotic foci (A–F). Immunofluorescence for CD68 (green, G and J), pAkt^S473^ (red, H), pAkt^T308^ (red, K) is shown co-localised (I and L) within macrophages in airspaces (panels G–L). In panels M–R immunofluorescence for pAkt^S473^ (red, N), pAkt^T308^ (red, Q) is shown localised to unstained cells morphologically characteristic of bronchial epithelium (N, Q, O and R). Cells morphologically characteristic of airway smooth muscle are evident (green, M and P). DAPI-stained nuclei are highlighted in blue. 25 μm scale bars are shown.

We first performed DAB-based immunohistochemistry (IHC) to explore evidence of functional PI3K signalling in areas of active fibrosis in IPF lung biopsy material (representative sections from n=12 patients shown), assessing the phosphorylation state of the critical downstream kinase, Akt. Online supplementary figure S1 shows prominent immunoreactivity for phosphorylated Akt^S473^ and Akt^T308^ localised to classic spindle-shaped, αSMA+ myofibroblasts and epithelium overlying fibrotic foci and cells morphologically characteristic of capillary endothelium (see online supplementary figure S1A,C,E). CD68+ macrophages residing in airspaces also exhibited signals for pAkt^S473^ and pAkt^T308^ (see online supplementary figure S1A,C,G). In contrast to myofibroblasts in fibrotic foci, airway smooth muscle cells demarcated by αSMA+ signal showed little evidence of Akt phosphorylation, while prominent phosphorylation was observed in the bronchial epithelium (see online supplementary figure S1B,D,F). A more detailed assessment of the cellular localisation of pAkt was provided by confocal dual immunofluorescence (figure 1). These studies confirmed the localisation of both pAkt^S473^ and pAkt^T308^ to αSMA+ myofibroblast subsets, however a differential staining pattern was observed in the overlying epithelium which preferentially exhibited signal for pAkt^S473^ (figure 1A–F). In the bronchial epithelium, pAkt^S473^ and pAkt^T308^ showed differential subcellular distribution, with pAkt^S473^ distributed apically to basolaterally throughout the cell layer, in comparison to pAkt^T308^ which was predominantly apically located (figure 1M–R). αSMA+ airway smooth muscle was negative for pAkt. CD68+ macrophages exhibited clear but heterogeneous staining pattern for pAkt^S473^ and pAkt^T308^ (figure 1G–L).

**Figure 2:**
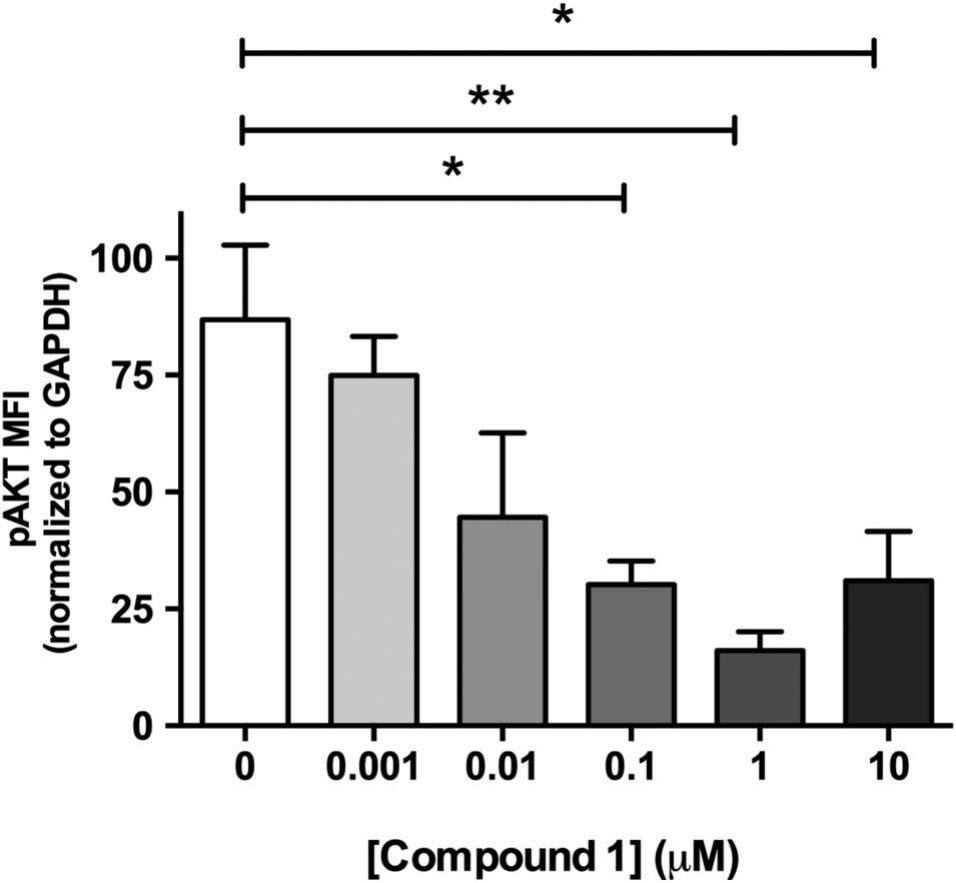
Pan-PI3 kinase (PI3K) dual mammalian target of rapamycin (mTOR) inhibition reduces Akt phosphorylation in human idiopathic pulmonary fibrosis (IPF) lung slices. Precision cut lung slices were treated with vehicle or increasing concentrations of PI3K inhibitor (Compound 1) for 2 h (0.1% DMSO vehicle was constant for all experimental conditions). Following harvest and homogenisation, pAkt levels were assessed by Milliplex Map transforming growth factor (TGF)-β signalling beads and normalised to glyceraldehyde 3-phosphate dehydrogenase (GAPDH). Data are shown for mean fluorescence intensity (MFI) normalised to GAPDH as ±SEM of n=4 technical replicate wells per condition; *p<0.05, **p<0.01 (one-way ANOVA with Tukey post-hoc testing).

Studies performed in live cultured precision-cut lung slices obtained from a patient with IPF confirmed prominent Akt phosphorylation in lung tissue and this signal was reduced in a concentration-dependent manner following incubation with a closely related analogue of GSK2126458, Compound 1 (figure 2) (see online supplementary figure S2 for compound structures and online supplementary table S1 for compound affinity data).

### GSK2126458 attenuates mitogenic responses in primary human LFs *in vitro*

**Figure 3:**
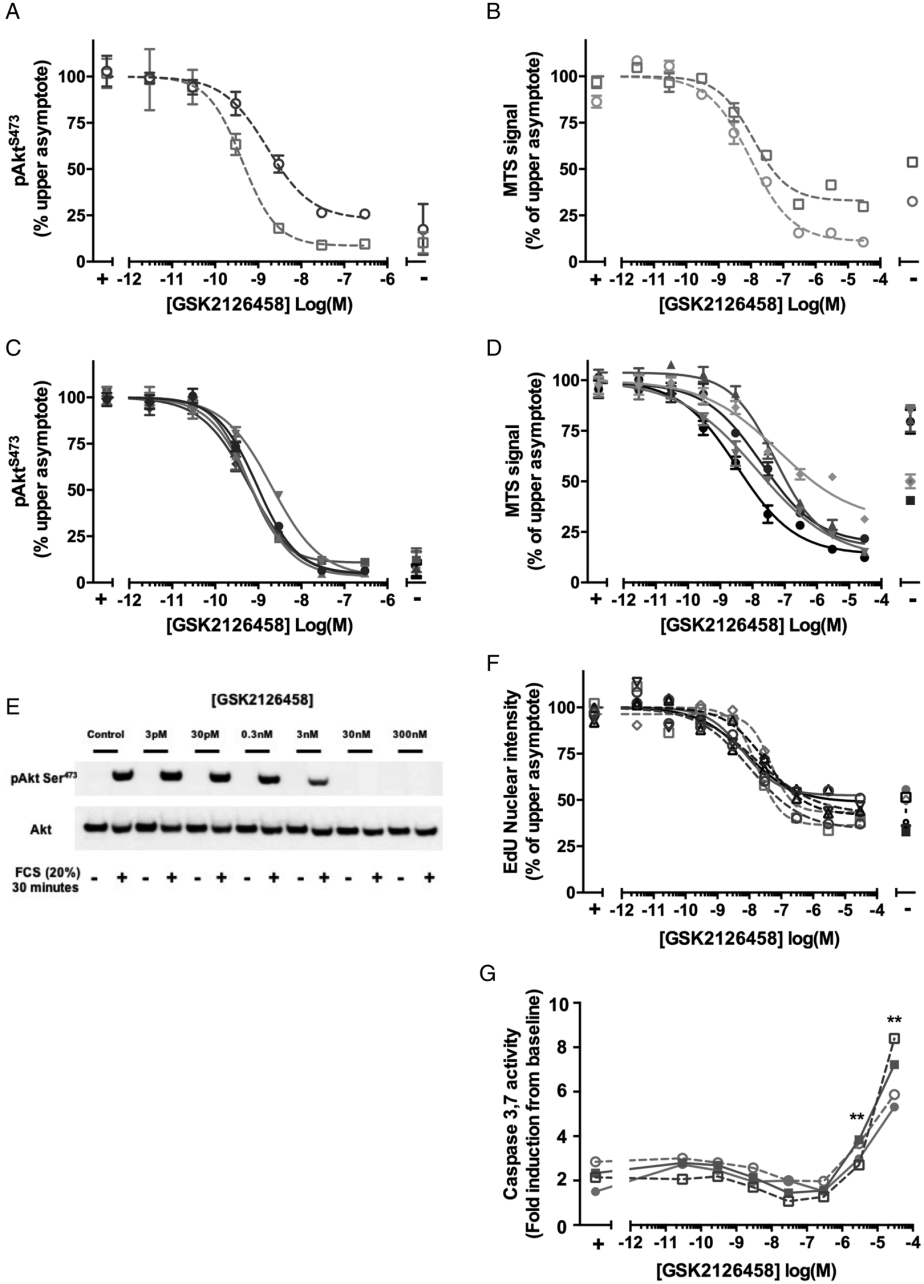
GSK2126458 inhibits Akt phosphorylation and proliferation in normal and idiopathic pulmonary fibrosis (IPF) primary human lung fibroblasts. Control (C-LF, n=2, A) or IPF lung fibroblasts (IPF-LF, n=5, C) were pre-incubated with increasing concentrations of GSK2126458. Following 30 min stimulation with 20% FCS, pAkt^S473^ signal was normalised to total Akt and expressed as % maximum response (maximum asymptote) and is shown as mean±SEM of n=3 replicate wells per condition. A representative Western blot is shown visualising the effect of GSK2126458 on FCS-induced phosphorylation of Akt^S473^ in a representative C-LF line (E). Subconfluent control (C-LF, n=2, B) or IPF lung fibroblasts (IPF-LF, n=5, D) were pre-incubated with increasing concentrations of GSK2126458. Following 72 h stimulation with 10% FCS, fibroblast proliferation was measured using an MTS assay at 490 nm. Data were expressed as % maximum response (maximum asymptote) and are shown as mean±n=6 replicate wells per condition. Incorporation of EdU (5-ethynyl-2′deoxyuridine) into the DNA of sub-confluent fibroblasts was also used as a measured of proliferation following 72 h of FCS stimulation (C-LF, n=5, dashed lines; IPF-LF, n=2, solid lines (F)). IC_50_ values (panels A–D and F) were calculated using four-parameter non-linear regression, and representative curves are shown for each fibroblast line. FCS stimulated and unstimulated DMSO vehicle control fibroblast data points are indicated as (+) and (−), respectively (panels A–F). DMSO was maintained at 0.1% for all conditions. Activation of fibroblast caspase 3 and 7 was assessed following incubation with increasing concentrations of GSK2126458 in the presence of 10% FCS by Caspase Glo (G). FCS-stimulated (+) vehicle control (0.1% DMSO) fibroblasts are indicated. Data are expressed as fold relative to baseline cells (no FCS, 0.1% DMSO) and shown as mean±SEM of n=3 replicate wells per condition. **p<0.0001 for differences in raw data values from FCS-stimulated controls (two-way ANOVA, Tukey's multiple comparisons test).

We next assessed the effect of GSK2126458 on Akt phosphorylation and proliferation of primary IPF and non-IPF control LFs (IPF-LF and C-LF). GSK2126458 inhibited serum-induced pAkt in C-LF (figure 3A and online supplementary table S2, geometric mean IC_50_=0.84 nM (range 0.43–1.64), n=2 donors) and IPF-LF (figure 3C and online supplementary table S2, geometric mean IC_50_=1.52 nM (95% CI 0.73 to 3.18), n=5 donors) in IPF-LF. Figure 3E shows a representative Western blot illustrating the effect of GSK2126458 on pAkt^S473^ phosphorylation. Figure 3B, D and online supplementary table S3 illustrate that GSK2126458 also inhibited FCS-induced fibroblast proliferation (C-LF geometric mean IC_50_=18.70 nM (95% CI 12.45 to 28.08, n=2 donors), IPF-LF geometric mean=23.64 nM (95% CI 13.61 to 41.07), n=5 donors) assessed by MTS assay. The inhibitory effects of GSK2126458 on FCS-induced fibroblast proliferation were confirmed using a second assay based on the incorporation of the modified thymidine analogue, EdU, into newly synthesised DNA (Click-iT EdU). The IC_50_ for GSK2126458 obtained in this assay system was comparable to that obtained in the MTS assay (figure 3F and online supplementary table S4, geometric mean IC_50_=15.91 nM; (95% CI 8.22 to 30.87)). Investigation of the effect of GSK2126458 on fibroblast apoptosis (induction of caspase 3,7 activity), showed that apoptosis was not detectable until concentrations ≥3 μM (figure 3G), indicating an approximately 25-fold window between the potent anti-proliferative effects of GSK2126458 and the induction of apoptosis in LFs.

### GSK2126458 inhibits Akt phosphorylation in IPF BALF cells

IHC studies (figure 1 and online supplementary figure S1) revealed that macrophages within IPF alveolar spaces were highly immunoreactive for pAkt. We therefore reasoned that since macrophages are a prominent cell type in IPF BALF, this observation could be exploited to develop a PD biosensor to monitor *in vivo* inhibition of PI3K signalling by GSK2126458.

**Figure 4:**
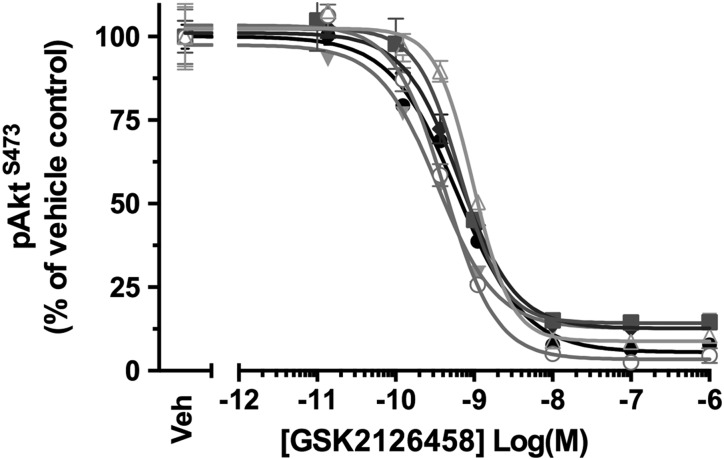
GSK2126458 inhibits Akt phosphorylation in bronchoalveolar lavage (BALF) cells from patients with idiopathic pulmonary fibrosis (IPF). IPF BALF cells (n=6) were incubated with 0.1% DMSO (vehicle control) or increasing concentrations of GSK2126458 for 30 min at 37°C (0.1% DMSO vehicle was constant for all experimental conditions). Cell lysates were assayed for total Akt and pAkt^S473^ using Meso Scale Discovery (MSD) electro-chemiluminescence technology. pAkt^S473^ signal was normalised to total Akt signal and expressed as % of a reference vehicle control well for each experiment (Veh). Data are shown as mean±SEM of triplicate wells per condition. IC_50_ values were calculated using four-parameter non-linear regression.

BALF differential cell counts confirmed that macrophages formed a prominent cell type (n=6, see online supplementary table S5). Akt phosphorylation was observed for BALF cells obtained for all patients and, incubation with GSK2126458 resulted in a concentration-dependent reduction in pAkt levels (figure 4 and see online supplementary table S6, geometric mean IC_50=_0.58 nM (95% CI 0.40 to 0.86)). The inhibitory profile of GSK2126458 was further found to be maintained in BALF cells re-suspended in ice-cold saline for up to 35 min following incubation with the drug (data not shown).

### Integrated PK/PD models to guide dose selection of GSK2126458 in patients with IPF

**Figure 5:**
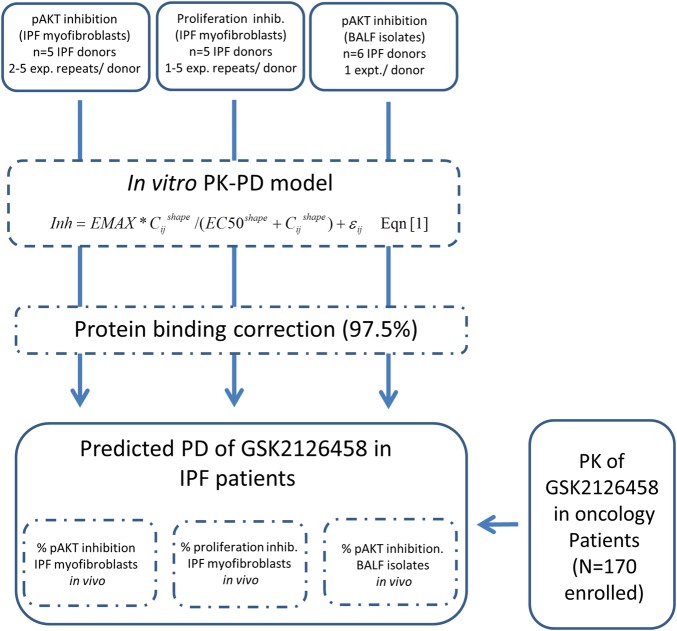
Construction of an integrated pharmacokinetic (PK)/pharmacodynamics (PD) model for GSK2126458 in patients with idiopathic pulmonary fibrosis (IPF). Schematic illustrating the strategy for integration of *in vitro* PK/PD data with human PK for clinical dose response estimation in IPF. Equation (1): Inh represents the % inhibition of pAkt (IPF myofibroblasts or bronchoalveolar lavage fluid (BALF) cell isolates) of fibroblast proliferation (IPF myofibroblasts), EMAX=maximum estimated inhibition, EC_50_=drug potency and shape=gradient of the response curve. ε refers to residual or unexplained variability, the subscript *i* indicates replicate cell line, *j* is individual drug concentration from each cell line.

To develop a dose rationale for GSK2126458 for future IPF clinical trials, the data from these *in vitro* studies were integrated with human PK data for GSK2126458 from ongoing clinical trials in patients with advanced solid tumours (http://www.gsk-clinicalstudyregister.com/study/112826#rs), according to the scheme outlined in figure 5. Stochastic model simulations were undertaken, providing predictions for pharmacological engagement over time for a range of doses. According to the model, the maximum tolerated dose of 2.5 mg twice a day dosing, GSK2126458 would likely provide enough exposure to significantly impact on the key PD endpoints, including Akt phosphorylation and fibroblast proliferation in the lungs of patients with IPF (table 1).

**Table 1:** Predicted pharmacodynamics of GSK2126458 in idiopathic pulmonary fibrosis patients by dose (Max: maximum inhibition (%), Min: minimum inhibition (%))

Dose (mg), twice daily	pAkt/Akt		Proliferation		BAL	
	Max	Min	Max	Min	Max	Min
0.25	14.3	8.3	24.5	19.2	24.2	12.4
1	35.8	23.2	36.4	30.4	62.5	45.7
2	49.6	34.9	42.5	37.1	76	65.2
2.5	54.1	38.7	44.6	39.6	79	69.7

BAL, bronchoalveolar lavage.

### Effect of GSK2126458 on epithelial cell apoptosis

**Figure 6:**
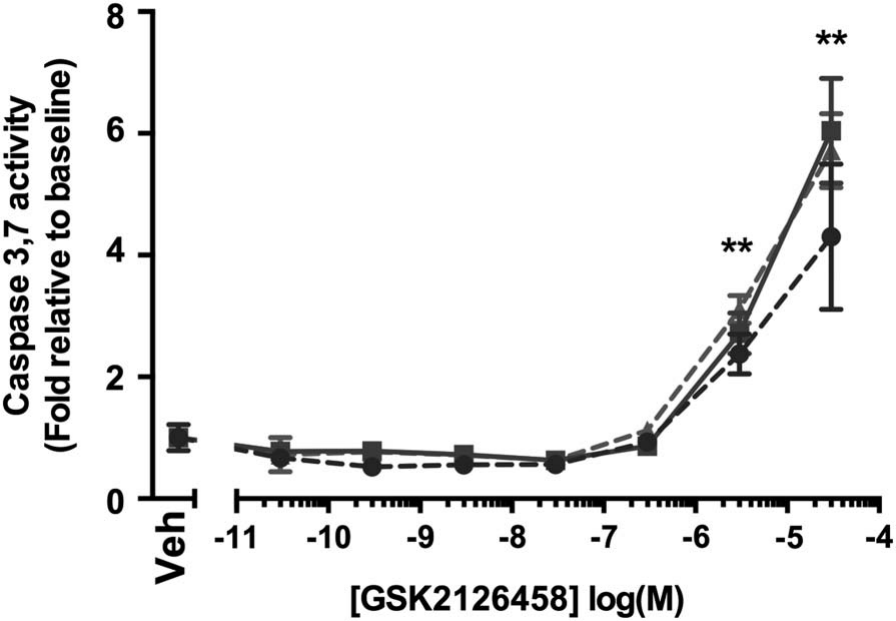
Effect of GSK2126458 on epithelial cell apoptosis. Activation of epithelial caspase 3 and 7 was assessed in primary bronchial epithelial cultures following incubation with increasing concentrations of GSK2126458 for 72 h (0.1% DMSO vehicle was constant for all experimental conditions). Human bronchial epithelial cell (HBEC) cultures were derived from n=2 control and n=1 idiopathic pulmonary fibrosis (IPF) donor lungs. Data are expressed fold relative to baseline (0.1% DMSO) controls (Veh) and shown as mean±SEM of n=6 replicate wells per condition; **p<0.0001 for differences in raw data values from no vehicle controls (two-way ANOVA, Tukey's multiple comparisons test).

The observation that the epithelium also displays a prominent pAkt signal lead us to further investigate the potential effect of GSK2126458 on epithelial cell apoptosis. Figure 6 illustrates that caspase 3,7 activity was induced in primary HBECs at concentrations of ≥3 μM (n=2 control donors, n=1 IPF donor).

### GSK2126458 inhibits pro-fibrotic responses to TGFβ_1_ in primary human LFs *in vitro*

Progressive, excessive and disorganised deposition of collagen and other extracellular matrix (ECM) proteins underlies the gross remodelling leading to loss of lung function in IPF, with TGFβ_1_ implicated as a critical mediator of this process.^5^ Recent reports have further suggested the involvement of the PI3K signalling axis in SMAD-independent TGFβ_1_ signalling.^15^ ^28^ We therefore also explored the potential of GSK2126458 to interfere with TGFβ_1_-induced fibroblast collagen production.

**Figure 7:**
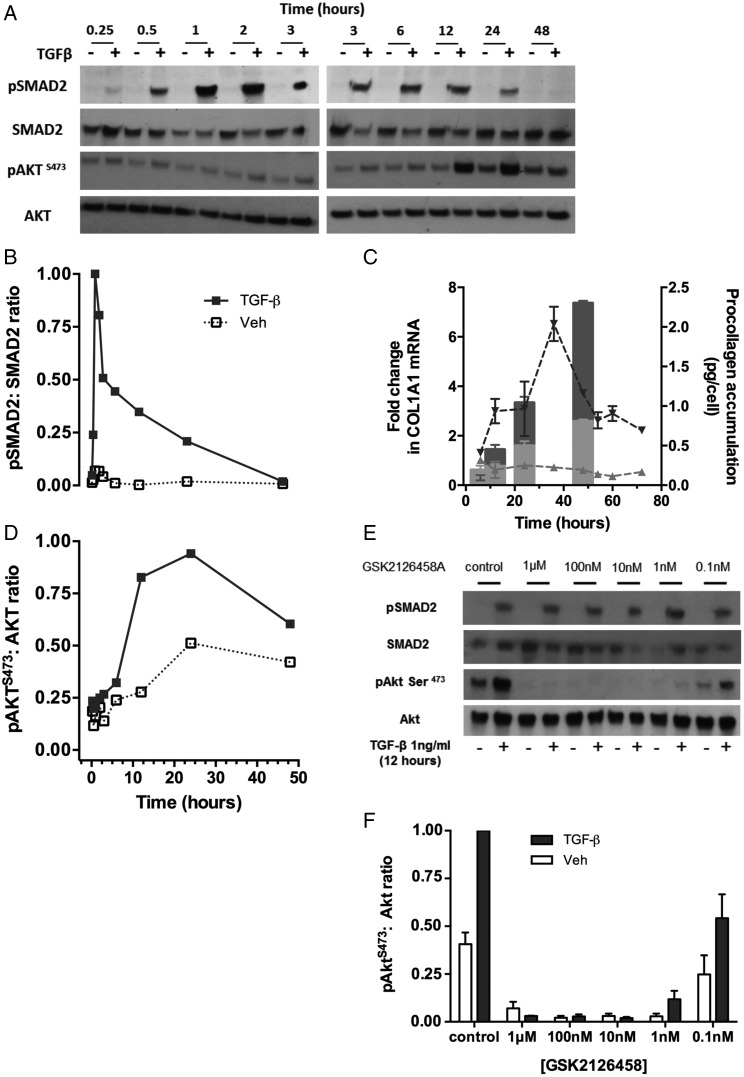
Time course of the primary human fibroblast response to transforming growth factor (TGF)-β_1_. Confluent lung fibroblasts (LFs) were stimulated with TGFβ_1_ (1 ng/mL) and lysates collected over the indicated timecourse (A, B and D). Phosphorylation of SMAD2 or Akt^S473^ is shown by Western blot (A) and densitometry (B and D). Collagen gene expression in TGFβ_1_ (1 ng/mL) stimulated (C, dark triangles and dashed line) or unstimulated (C, light triangles and dashed line) was assessed in LFs over the indicated time course. Pro-collagen recovered from cell supernatants over the same time course in TGFβ_1_ stimulated (C, dark bar) or unstimulated (C, light bar) is also shown. Serum-free confluent primary human LFs (HLFs) were incubated with increasing concentrations of GSK2126458 or 0.1% DMSO and stimulated with TGFβ_1_ (1 ng/mL) for 12 h (0.1% DMSO vehicle was constant for all experimental conditions). Phosphorylation of SMAD2 and Akt^S473^ is shown by representative Western blot (E). Densitometry illustrating the effects of GSK2126458 on pAkt^S473^ in HLFs from n=3 donors is shown (F).

We first defined the kinetics of PI3K activation in relation to canonical SMAD signalling following TGFβ_1_ (1 ng/ml) stimulation. Figure 7A, B illustrates that exogenous TGFβ_1_ induces rapid phosphorylation of SMAD2 peaking at 2 h, with pAkt^S473^ exhibiting a slower kinetic peaking at 12–24 h (figure 7D). Delayed Akt phosphorylation is in agreement with previous reports, suggesting the potential involvement of an autocrine mediator.^15^ Of note in our experiments, this peak precedes the peak of collagen (*COL1A1*) gene expression (36 h; figure 7C).

We next assessed the effect of GSK2126458 on SMAD2 and Akt phosphorylation at 12 h after the addition of exogenous TGFβ_1_. Figure 7E shows that GSK2126458 had no influence on SMAD2 phosphorylation but inhibited TGFβ_1_-induced Akt phosphorylation in a concentration-dependent manner, with maximum inhibition obtained at 10 nM (figure 7E,F).

**Figure 8:**
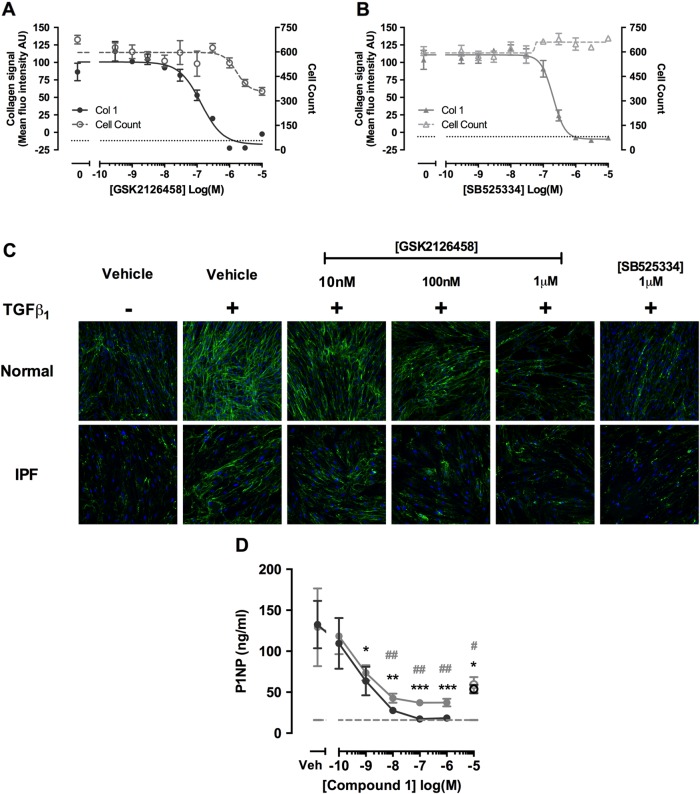
Pan-PI3 kinase (PI3K)/mammalian target of rapamycin (mTOR) inhibition attenuates transforming growth factor (TGF)-β_1_-induced collagen production in human lung fibroblasts (LFs), and collagen formation markers in idiopathic pulmonary fibrosis (IPF) lung tissue. Confluent LFs were incubated with increasing concentrations of GSK2126458 (A) or SB525334 (B) and stimulated for 48 h with TGFβ_1_ (1 ng/mL) and collagen biosynthesis assayed by molecular crowding assay (0.1% DMSO vehicle was constant for all experimental conditions). Data are expressed as mean fluorescent intensity (n=4 reads per well) and cell counts obtained from DAPI counterstaining (blue channel). Data are shown as mean±SEM of n=3 replicate wells per condition. IC_50_ values were calculated using four-parameter non-linear regression. Replicate experiments were carried out on human lung fibroblasts (HLFs) from a non-IPF donor and representative curves are shown. Images from each cell treatment are shown (C). 8 mm slices of IPF lung were cultured for 24 h in DMEM supplemented with 0.4% FCS and treated with vehicle, 10 μM SB525334 (open circles) or increasing concentrations of Compound 1 (inhibitor) for 0–3 days (filled grey circles) and further from 3 to 5 days (filled black circles) following media change. P1NP levels were measured in harvested supernatants (D). Data are shown as ±SEM of n=4 replicate wells per condition; *p<0.05, **p<0.01 (two-way ANOVA with Bonferroni post-hoc testing).

We next characterised the potency of GSK2126458 for inhibiting TGFβ_1_ induced collagen deposition in myofibroblasts, using a novel high-content imaging based molecular crowding assay.^27^ Figure 8A illustrates that GSK2126458 attenuated TGFβ_1_-induced collagen deposition in fibroblasts, with an IC_50_ of 132.5 nM (95% CI 65.5 to 268), and leads to a reduction in cell number with an IC_50_ of 1.66 μM (95% CI 0.51 to 5.43). Induction of caspase 3,7 in molecular crowded conditions is consistent with concentrations of GSK2126458 which cause reduction in cell count, suggesting apoptosis in crowded conditions may be responsible for the observed reduction in cell number (see online supplementary figure S3). Collectively these data indicate a marked separation between the inhibitory effects of GSK2126458 on collagen deposition and induction of apoptosis. For comparison, the selective TGFβRI (ALK5) inhibitor, SB525334, inhibited TGFβ_1_ induced collagen deposition with an IC_50_ of 200.5 nM (95% CI 159 to 252; figure 8B). Images capturing the effects of inhibitors on primary fibroblasts isolated from IPF and control lungs in this assay are also shown (figure 8C).

### Pan-PI3K/mTOR inhibition reduces collagen formation markers in ex vivo slices of IPF lung tissue

Our final aim was to explore evidence for a direct functional link between PI3K/mTOR signalling and matrix formation in this disease setting by monitoring the release of the human pro-collagen 1 amino-terminal peptide (P1NP) in IPF tissue slices using a competitive ELISA based on a proprietary monoclonal antibody.^25^ Figure 8D shows that the related GSK2126458 analogue, Compound 1, reduced P1NP levels released into lung slice supernatants, with maximal inhibition observed at >100 nM; the effect of a Alk5 inhibitor, SB525334 (10 µM), is also shown.

## Discussion

PI3K represents a key oncogenic signalling node, implicated in a range of cellular processes from inflammation to cancer. More recent evidence also suggests a role for this pathway in fibrosis, however to the best of our knowledge there are currently no PI3K agents in clinical development for IPF or any other fibrotic condition. The aim of the current study was to further explore the rationale for targeting PI3K signalling in IPF and to define the pharmacological profile of a potent and clinically advanced PI3K/mTOR inhibitor, GSK2126458, as a novel anti-fibrotic.

PI3K mediates the conversion of phosphatidylinositol 4,5 bisphosphate (PIP2) to phosphatidylinositol 3,4,5 trisphosphate (PIP3) at the plasma membrane, inducing the recruitment and phosphorylation of Akt at threonine 308 (pAkt^T308^) and serine 473 (pAkt^S473^) to initiate downstream signalling. The initial focus for our PI3K pathway validation studies in IPF was therefore to perform detailed IHC studies to determine Akt phosphorylation in IPF biopsy tissue. These studies showed demonstrable immunoreactivity for pAkt^S473^ associated with activated myofibroblasts within IPF fibrotic foci, as previously suggested.^18^ We further provide evidence for pAkt^T308^ immunostaining associated with these myofibroblasts and thus provide the first evidence of full Akt activation in situ in this disease context. Immunofluorescent confocal co-localisation studies further provide the first evidence of cell-specific differential phosphorylation of Akt: whereas pAkt^T308^ immunostaining predominates in myofibroblasts and a thin band of cells located at the epithelial–mesenchymal border of fibrotic foci, pAkt^S473^ immunostaining was evident in both the epithelium and myofibroblasts. Moreover, heterogeneous distribution and phosphorylation of Akt were observed in bronchial epithelium and macrophages in the IPF lung. Differential phosphorylation of Akt has not been extensively studied; however in cancer, it has been proposed to influence the interactions of Akt with its substrates and thereby influence cell motility and invasion.^29^ ^30^ Of interest, in non-small cell lung cancer, elevated pAkt^T308^ relative to pAkt^S473^ is associated with poor prognosis.^31^ Future studies to determine the implications of differential phosphorylation of Akt in the context of IPF are beyond the scope of this article but may shed important light on the role of Akt in this disease setting. Confidence in PI3K as a target pathway was further extended by functional studies in IPF-derived lung tissue. Akt phosphorylation was readily detectable in precision-cut cultured IPF lung slices and this signal was inhibited by pan-PI3K/dual mTOR inhibition in a concentration-dependent manner.

Subsequent proliferation studies revealed that GSK2126458 inhibited serum-induced Akt phosphorylation and proliferation at nanomolar potency with no difference in compound potency observed. Of note, this anti-proliferative profile of GSK2126458 is comparable to that observed in previous *in vitro* oncology studies using the BT474 and T47D breast cancer lines.^21^

While pan-PI3K/mTOR inhibitors offer the advantage of overcoming functional redundancy between class I isoforms,^32^ and blocking potential crosstalk and feedback of compensatory mechanisms through inhibition of three key nodes (PI3K, mTORC1 and mTORC2), the prospect for mechanism-based on-target toxicities for this class of inhibitor, in particular with respect to inducing apoptosis, are well documented.^33^ In our studies, using primary LFs and bronchial epithelial cells, the anticipated pro-apoptotic effects associated with inhibiting PI3K/mTOR signalling were not manifest until concentrations of GSK2126458 were significantly higher than those required for exerting demonstrable anti-fibroproliferative and pro-apoptotic effects. This window is encouraging and suggests there is considerable potential for GSK2126458 to be administered at concentrations that would primarily impact on deleterious fibroblast functional responses in IPF.

Assessment of drug PDs in the lung of patients with IPF is challenging due to limitations associated with tissue access. Our IHC observation of pAkt immunostaining being associated with macrophages within airspaces raised the possibility that cells harvested from IPF BALF could serve as a potential PD biosensor for monitoring effective pharmacological engagement of mechanism; namely, pAkt inhibition in the lungs of patients recruited to early PoM studies. To this end, a BALF cell processing protocol was developed which involved minimal cell handling by assaying pAkt^S473^ in whole BALF cell pellets on the basis that macrophages were a prominent cell type present. These data would also provide additional evidence from primary patient-derived lung cells to further support a dose rationale for GSK2126458. Interestingly, BALF cell pellets from patients with IPF exhibited pAkt^S473^ activation without the need for exogenous stimulation and were sensitive to GSK2126458 inhibition, at sub-nanomolar concentrations. Although inhibiting macrophage function was not the major basis for exploring GSK2126458 as a potential novel treatment for IPF, a potential role for macrophages, specifically alternatively activated M2 macrophages, is becoming increasingly recognised.^34–36^ Recent studies have implicated PI3K signalling in promoting M2 polarisation in IPF-derived BAL macrophages,^37^ raising the possibility that modulating macrophage biology might itself provide an additional benefit to GSK2126458 treatment in this disease context.

To build a model for dose prediction in IPF, pharmacological data obtained from fibroblast signalling and mitogenic assays were integrated with BALF cell PI3K phosphorylation data and existing PK data from previous human oncology trials. The model simulations predicted that within the typical clinical dose range (2–3 mg twice daily), GSK2126458 would significantly impact fibroblast PI3K signalling and proliferation in the lungs of patients with IPF.

The decision to progress GSK2126458 to a PoM trial in IPF was predicated on an appropriate dose rationale for this compound as a fibroblast anti-proliferative agent, based on the widespread evidence linking this pathway to cell growth and proliferative responses in multiple cell types and disease settings.^38^ However, in terms of key IPF pathomechanisms, we were also interested in determining the potential of this agent to inhibit TGFβ_1_-induced myofibroblast collagen production, based on limited literature reports suggesting the involvement of PI3K in SMAD-independent TGFβ_1_ signalling.^15^ ^28^ These studies revealed that GSK2126458 was highly effective at attenuating TGFβ_1_-induced myofibroblast collagen deposition at concentrations around the 100 nM range.

Our final aim was to directly link PI3K/mTOR signalling to pro-fibrotic collagen formation in IPF lung tissue using an ex vivo human tissue model. Type I collagen is the most represented extracellular protein in the lungs and its dysregulated turnover is a hallmark of fibrosis. During maturation of type I collagen, soluble pro-collagen is processed to its insoluble form by carboxy and amino terminal endopeptidase cleavage, generating peptide fragments such as the N-terminal P1NP which can be monitored as a sensitive biomarker of collagen formation.^25^ Our studies revealed that P1NP was released from ex-vivo IPF precision-cut lung slices for up to 5 days in culture and further that PI3K/mTOR inhibition attenuated this process. To the best of our knowledge, these studies represent the first direct demonstration of a direct functional link between PI3K/mTOR signalling and ECM formation in this human disease setting. It is noteworthy that in isolated fibroblasts and in lung tissue slices, the pan-PI3K/mTOR inhibitors exhibited comparable efficacy to the selective TGFβRI (Alk-5) inhibitor, SB525334. This raises the possibility that GSK2126458 could interfere with the fibrogenic effects of TGFβ_1_ signalling at clinically attainable doses, while potentially sparing its essential homeostatic and tumour suppressor functions. This is a particularly important consideration in patients with IPF who are at a heightened risk of developing lung cancer.^5^ ^39^

In conclusion, we have considerably extended the scientific rationale for targeting PI3K signalling in IPF using patient-derived cells and lung tissue. We provide evidence for active PI3K signalling in the fibrotic foci of IPF patients together with data that GSK2126458 attenuates serum-induced proliferation and TGFβ_1_-induced collagen production in IPF fibroblasts. Population modelling and integration of preclinical biomarker data with human PK data has enabled us to develop a dosing framework which should safely and effectively engage PI3K signalling in the lungs of patients with IPF, providing support for progressing GSK2126458 to further clinical evaluation in IPF in a dose-finding, double-blind, placebo-controlled study (https://clinicaltrials.gov/ct2/show/NCT01725139). To the best of our knowledge, this represents the first IPF clinical study aimed at assessing target engagement and tolerability of a PI3K/mTOR inhibitor in this disease setting.
