# Supplementary material for: Exploration of a potent PI3 kinase/mTOR inhibitor as a novel anti-fibrotic agent in IPF
Source: Thorax. 2016 Apr 21;71(8):701–11. doi: 10.1136/thoraxjnl-2015-207429 (PMC4975851; doi:10.1136/thoraxjnl-2015-207429)
Supplement: Supplementary data [file thoraxjnl-2015-207429supp.pdf]

Supplementary Material

Immunohistochemistry for light microscopy: Antigen unmasking was achieved by microwaving in Access Supreme buffer (pH 6.2, A. Menarini diagnostics) for αSMA and CD68 or 10mM citrate buffer (pH 6) for pAkt^{Ser473} and pAkt^{Thr308}. Antibodies against αSMA (Dako; M0851, 0.047μg/ml, 1:1500 dilution), CD68 (Dako; M0876, 0.06μg/ml, 1:500 dilution), pAkt^{Ser473} (Epitomics; 2118-1, 2.7μg/ml) and pAkt^{Thr308} (Abcam; ab38849, 5μg/ml) were used. Sections were digitally scanned with a Hamamatsu NanoZoomer and representative images are presented.

Immunohistochemistry for Confocal microscopy: Antibodies against pAkt^{S473} (1:50) and pAkt^{T308} (1:50) were co-localized with αSMA (1:500) and with CD68 (1:200) using a standard ABC enhanced indirect IHC approach. Primary αSMA and CD68 antibodies were labelled with secondary Alexa Fluor® 488 anti-mouse antibodies (Life Technologies; A11001, 1:200) and primary pAkt antibodies with secondary biotinylated anti-rabbit antibodies (VectorLab; BA-1000, 1:200) subsequently enhanced with Alexa Fluor® 546 conjugated streptavidin-biotin complex (Life Technologies; S-11225, 2mg/mL, 1:400). Digitised images were captured on a Leica DM6000 CS microscope fitted with a Leica TCS SP8 confocal head and the Leica LAS-X software suite (Leica Microsystems GmbH) and finally prepared with ImageJ.

Primary fibroblast culture. Primary human lung fibroblasts (LFs) were grown from explant cultures of IPF (IPF-LF) or non-IPF control (C-LF) lung tissue, grown on tissue culture plastic (NUNC™, Thermo Scientific) as previously described[1]. Briefly,

lung tissue biopsies were cut into 1-mm^3 cubes and cultured in Dulbecco's modified Eagle's medium supplemented with 10% (v/v) newborn calf serum, penicillin (100 U/ml), streptomycin (100 μg/ml), and 2.5 μg/ml amphotericin B (all from Thermo Fisher Scientific). A near confluent monolayer of fibroblasts was obtained after 3 to 4 weeks and passaged. Experiments were conducted on cells between passages 3 and 13. Fibroblast cell line purify was confirmed by immunohistochemical characterization using antibodies to cytokeratin, von Willebrand factor, and desmin to rule out contamination by epithelial, mesothelial, endothelial, or smooth muscle cells. All lines generated were negative for these markers and > 95% of cells stained positively for the mesenchymal marker, vimentin. Between 20 and 30% of cells were also positive for myofibroblast marker, α-smooth muscle actin. LFs were used between passage 3 and 13, maintained in DMEM at 37°C, 10% CO_2, 100% humidity supplemented with L-glutamine, penicillin, streptomycin, fungizone, and 10% FBS (all from Thermo Fisher Scientific). For most experimental series LFs were seeded to become confluent within 24 hours prior to 24 hour serum starvation. The exception to this was for proliferation experiments where LFs were seeded at 7500 cells per well (96 well format), to allow a window for proliferation.

Primary human bronchial epithelial cell culture. Primary human bronchial epithelial cells (HBECs) were obtained from regions of normal airway of IPF or non-IPF control lung tissue as previously described[2]. HBECs were used at passage 3 and 4, maintained in BEGM (Lonza, Switzerland) at 37°C, 5% CO_2, 100% humidity.

HBECs apoptosis. Sub-confluent HBECs were incubated for 72 hours in BEGM containing 10% FCS and 0.1% DMSO or increasing concentrations inhibitor.

Caspase 3/ 7 activation in HBECs indicative of apoptosis was measured using Caspase Glo (Promega), as per manufacturer's instructions.

Fibroblast Akt signalling. Serum starved confluent LFs were incubated for 30 minutes (10% CO2, 100% humidity) in DMEM containing 20% FCS and either 0.1% DMSO or increasing concentrations of inhibitor. Cells were lysed in ice cold PhosphoSafe® buffer (Millipore). Cell lysates were assayed for total Akt and $pAkt^{S473}$ using either the Meso Scale Discovery (MSD) system or Sodium dodecyl sulphate polyacrylamide gel electrophoresis and Western blot.

Western blot. Following SDS PAGE and blocking, membranes were incubated with antibodies to detect $pSMAD2^{Ser465/467}$ (#3101, Cell Signalling Technology), or $pAkt^{Ser473}$ (#4060, Cell signalling Technology), and signals visualized with ECL Western Blotting Detection Reagents (GE Healthcare). Following stripping, membranes were blocked and re-probed with antibodies specific for total SMAD2 (#3103, Cell Signalling Technology) or total Akt (#4060, Cell Signalling Technology).

pAkt signalling in BALF cell pellet. BALF cell pellets were prepared by centrifugation (300g, 5 minutes) and differentially counted. Following re-suspension cells were incubated in RPMI containing 0.1% DMSO (vehicle control) or increasing concentrations of GSK2126458 (30 mins, 37 ^{o}C, 5% CO_2, 100% humidity). Following incubation suspensions were centrifuged (300g, 5 minutes) and cell pellets lysed in ice cold PhosphoSafe® buffer (Merck Millipore). Cell lysates were assayed for total Akt and $pAkt^{S473}$ using the Meso Scale Discovery (MSD) system.

Fibroblast proliferation. LFs seeded at a density of 7500 per well were serum starved for 24 hours and subsequently incubated for 72 hours in DMEM containing 20% FCS and 0.1% DMSO or increasing concentrations inhibitor. Proliferation was assayed using CellTiter 96 Aqueous (Promega) or using the Click-iT® EdU Alexa Fluor® 488 Imaging Kit (ThermoFisher Scientific) as per manufacturer's instructions. Caspase 3/ 7 activation in LFs indicative of apoptosis was measured under identical conditions using Caspase Glo (Promega), as per manufacturer's instructions.

Col1A1 RT-PCR. Real time RT-PCR was conducted using the MESA GREEN qPCR MasterMix Plus for SYBR® Assay (Eurogentec). 2µl of cDNA and forward and reverse primers were added at a final concentration of 800nM (Col1A1 forward primer: 5'-ATGTAGGCCACGCTGTTCTT-3'; Col1A1 reverse primer: 5'-GAGAGCATGACCGATGGATT-3'. Samples were run as duplicates on the Mastercycler EP Realplex (Eppendorf, Germany). Cycling conditions: 95ºC for 10 minutes for SYBR green activation, 95ºC for 5 seconds and 60ºC for 45 seconds for 40 cycles. Col1A1 crossing point (Cp) values were determined from the linear region of the amplification plot and normalized by subtraction of the geometric mean of the crossing point (Cp) values for two housekeeping genes: ATP synthase 5B (*ATP5B*) and β2 macroglobulin (B2M), identified by GeNorm analysis as the most stable housekeeping genes for this study. Statistical analysis was performed on ΔCp values. Fold change relative to time zero was subsequently calculated using the standard $2^{-\Delta\Delta Cp}$ approach. All primers and GeNorm kits (Primer Design).

Preparation of precision cut lung slices. IPF lung tissue was inflated with 3%

agarose (Sigma), 8mm cores prepared and 250µm slices generated using a Krumdieck tissue slicer and maintained overnight in DMEM at 37°C, 10% CO_2, 100% humidity supplemented with L-glutamine, penicillin, streptomycin, fungizone, and 10% FBS (all from Invitrogen). For experiments, medium was replaced with fresh DMEM (0.4%FCS) with 0.1% DMSO or increasing concentrations inhibitor for 2 hours at 37°C. Lung slices were homogenized in ice cold PhosphoSafe® buffer (Millipore) and assayed for pAkt using the Milliplex TGFβ signalling bead assay (Millipore; 48-614MAG), normalized to multiplexed GAPDH beads (Millipore; 46-667)."

In separate experiments supernatants were harvested for assessment of P1NP by proprietary competitive ELISA as previously described[3].

Building a dose prediction model for GSK2126458 in IPF.

All *in vitro* data were first analyzed as a naïve pool using a non-linear mixed effects model[4] as a simulation of the inter-donor variability for fibroblast and BALF cell PD. Using this approach, the cell assay data were described by the sigmoidal relationship described in Eqn. 1, Figure 5 (for output parameter estimates see Table 1). Next, predicted plasma concentration profiles for GSK2126458 in IPF patients were generated based on population PK data obtained from on-going oncology studies with GSK2126458 in patients with advanced solid tumours (http://www.gsk-clinicalstudyregister.com/study/112826#rs). The modelled PD and predicted PK data were combined in a stochastic simulation to predict target engagement in IPF patients at twice daily doses of 0.25, 1, 2 and 2.5 mg.

Determination of collagen deposition by molecular crowding assay. Collagen biosynthesis in 96 well format was measured by a high-content imaging based molecular crowding assay modified from a previously described method[5]. Briefly, confluent LFs were cultured in DMEM containing 0.4% FCS and ascorbic acid (100μM), in the presence of mixed Ficoll 70 and Ficoll 400 as molecular crowding agents. These were stimulated with $TGF\beta_1$ (1ng/ml) and incubated with either vehicle (0.1%DMSO) or increasing concentrations of GSK2126458 or SB525334 for 48 hours. LFs were fixed and stained with antibody specific for human collagen 1, fluorescent secondary antibody (Alex Fluo488) and nuclei counterstained with DAPI for per cell normalisation. Fluorescent signal quantified on the INCELL 6000 high content system.

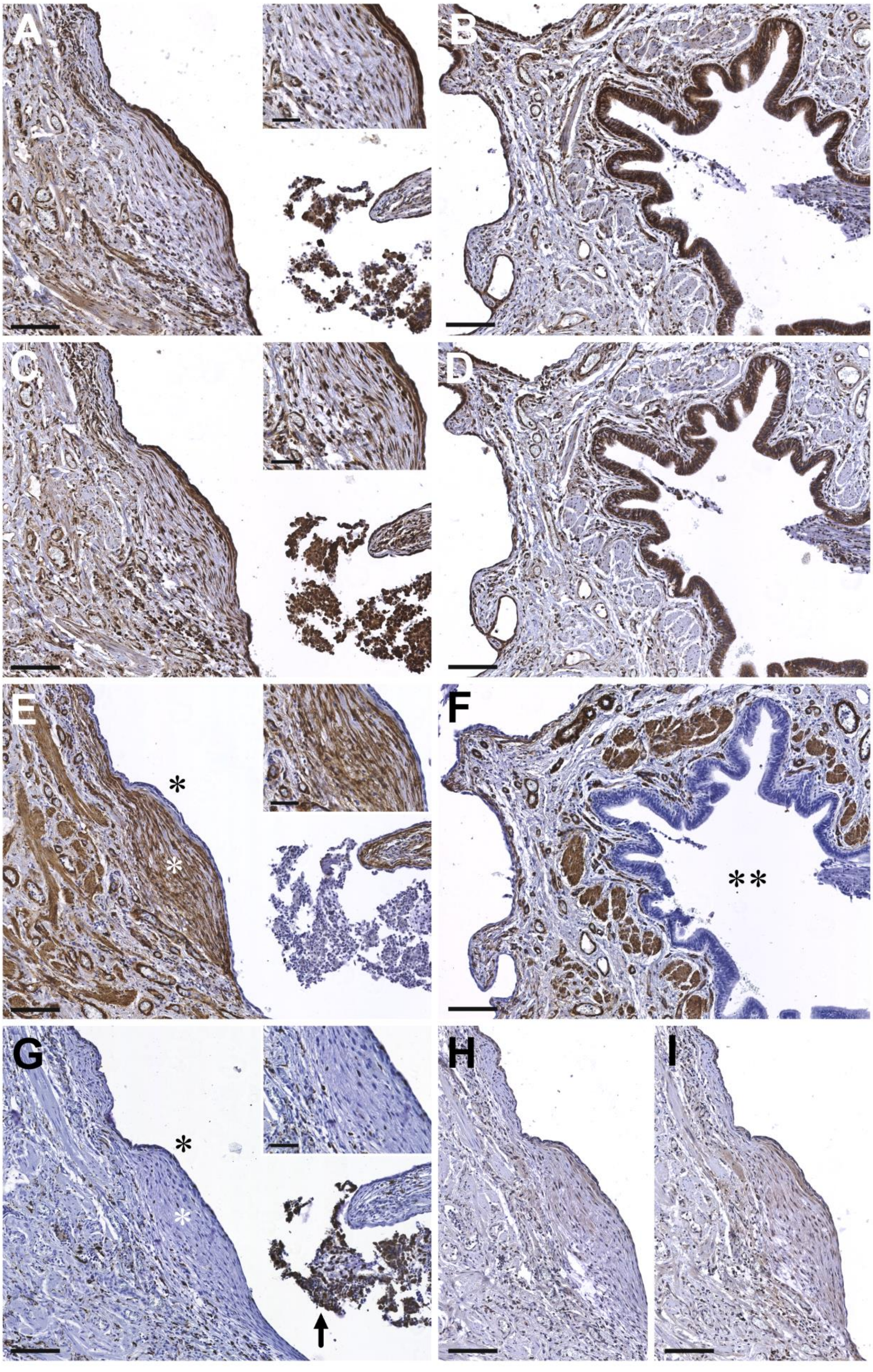
A
B
C
D
E
F
G
H
I

Figure S1. Akt phosphorylation with fibrotic foci in the IPF lung. Immunoreactivity for Akt phosphorylated at serine 473 (pAkt^{S473}, **A**) and threonine 308 (pAkt^{T308}, **C**) is shown in serial sections of discrete fibrotic foci, localizing to spindle shaped cells immuno-reactive for αSMA (white asterisk, **E**), as well as capillary endothelium. Immuno-staining for both phosphorylation sites was also observed in epithelial cells overlying the fibrotic focus (pAktS473 **A** and pAktT308 **C,** black asterisk **E**). Signal for pAktS473 and pAktT308 was present in bronchial epithelium (**B, D** respectively), and macrophages delineated by CD68 (Arrow, **G**; pAktS473, **A**; pAktT308,**C**), with poor signal pAkt signal observed in αSMA positive airway smooth muscle (**F,** pAktS473, **B**; pAktT308,**D**); scale bar, 150μm. The fibrotic focus is also shown at high power, scale bar 50μm (inset) Negative immuno-staining with non-immune IgG at equivalent dilutions to pAkt^{S473} and pAkt^{T308} specific antibodies also shown (**H** and **I**).

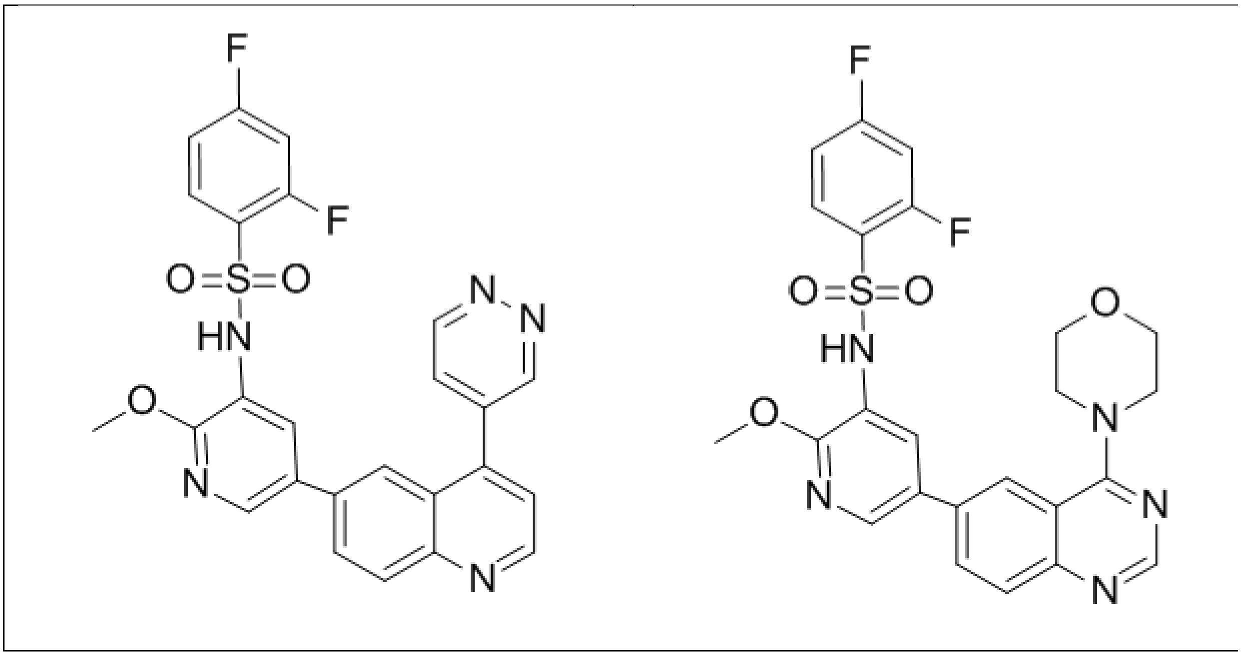

Figure S2. Chemical structures for GSK2126458 (A) and Compound 1 (B)

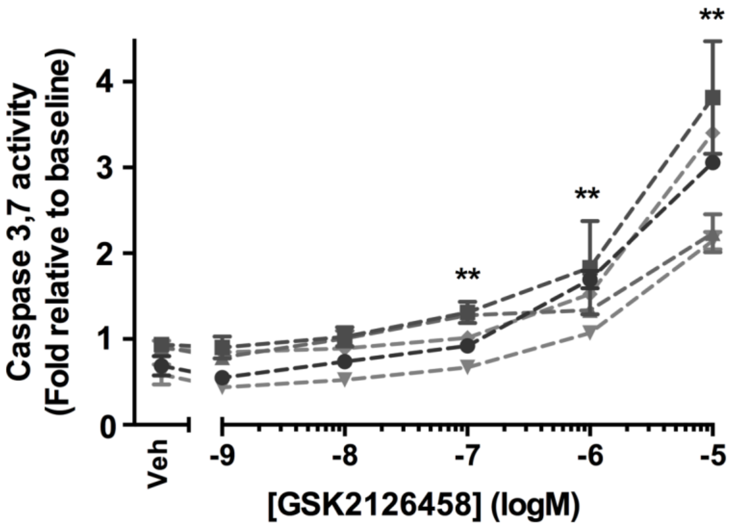

Figure S3. Induction of Apoptosis in crowded cell culture. Activation of fibroblast caspase 3 and 7 was assessed in confluent crowded culture conditions following TGFβ stimulation and treatment with increasing concentrations of GSK2126458. Data from n=5 C-LF lines are shown and data expressed fold relative to baseline without TGFβ (0.1%DMSO) (Veh) and shown as mean +/- SEM for n=4 replicate wells per condition; ** *P*<0.0001 for differences in raw values from no vehicle controls (2 way ANOVA, Tukey's multiple comparisons test).

	**Compound 1**			**GSK2126458**		
	**pIC50**	**n (total)**	**Comments**	**pIC50**	**n (total)**	**Comments**
PI3K-alpha	10.4	3	2 values of > 9.9	9.5	10	2 values of > 10.0
PI3K-beta	9.5	3	1 value of > 9.9	9.1	10	
PI3K-delta	10.6	3	2 values of > 9.6	9.3	10	5 values of > 9.58
PI3K-gamma	9.9	2	1 value of > 9.4	9.3	10	
FRAP1	9.5	1		9.6	1	

Table S1. pIC_{50} for PI3K/ mTOR inhibitors in recombinant assays (GSK). pIC_{50} in recombinant assays (GlaxoSmithKline, Upper Merion, PA). Data points below the concentration limit of the assay are excluded from the summary mean in the PI3K isoform data. An overview of the data is provided in the table. The pIC50 quoted is the mean for the non-modified values; the n is the total of instances the compound has been assayed and the comment field illustrates the instances when the compound tested beyond the limit of the assay.

pAkt summary pharmacology

A

**Control fibroblasts**	Passage	IC50 (nM)	Hill Slope
Donor 1	6	0.43	-1.08
Donor 2	11	1.64	-0.84
	Geo mean	0.84	
	range	0.43-1.64	

B

**IPF fibroblasts**	Passage	IC50 (nM)	Hill Slope
Donor 1	7	0.64	-0.79
	10	2.31	-0.72
Donor 2	5	0.58	-1.08
	6	0.39	-0.59
	7	13.71	-0.69
	10	14.09	-1.68
Donor 3	8	1.67	-2.31
	9	0.94	-0.92
	13	1.02	-1.12
Donor 4	6	0.68	-0.89
	7	1.04	-1.24
Donor 5	13	2.12	-0.78
	Geo mean	1.52	
	95% CI	0.73- 3.18	

Table S2: Summary pharmacology of the effects of GSK2126458 on pAkt in fibroblasts

MTS summary pharmacology

A

**Control fibroblasts**	Passage	IC50 (nM)	Hill Slope
Donor 1	6	9.75	0.66
	7	53.951	-0.51
	8	11.376	-0.46
	9	39.174	-0.84
	10	22.387	-0.83
Donor 2	5	18.239	-0.63
	6	11.588	-0.80
	7	15.101	-0.56
	8	25.235	-0.99
	9	12.359	-0.88
	Geo mean	18.70	
	95% CI	12.45- 28.08	

B

**IPF Fibroblasts**	Passage	IC50 (nM)	Hill Slope
Donor 1	8	65.31	-0.37
	9	29.79	-1.03
	10	43.95	-0.62
Donor 2	7	21.98	-0.56
	8	66.07	-0.74
	9	19.41	-0.50
	5	19.36	-0.70
	6	9.98	-0.60
Donor 3	8	3.46	-0.48
	11	71.12	-0.35
Donor 4	6	51.05	-0.63
	7	9.98	-0.60
Donor 5	13	12.36	-0.99
	Geo mean	23.64	
	95% CI	13.61- 41.07	

Table S3: Summary pharmacology of the effect of GSK2126458 on pHLF proliferation (MTS)

EdU proliferation summary pharmacology

**Control fibroblasts**	Passage	IC50 (nM)	Hill slope
Donor 1	6	10.53	-0.53
Donor 2	6	32.51	-0.69
Donor 3	8	17.59	-0.46
Donor 4	7	17.32	-1.17
Donor 5	7	46.31	-1.31
Donor 6 (IPF)	3	6.43	-0.77
Donor 7 (IPF)	10	8.32	-0.57
	Geo mean	15.91	
	95% CI	8.22- 30.87	

Table S4: Summary pharmacology of the effect of GSK2126458 on pHALF proliferation (EdU)

Donor	Differential cell count on IPF BALF isolates (% total)					Total
	Mac	Lymph.	Neut.	Eosin.	Mast.	(x10^3 cells/ml)
1	72	13.7	5	3	0	94
2	58.7	21	5	12	1.7	214
3	73.7	7	7	7	0	146
4	76	7	9	8	0	312
5	51.3	12	28.3	6.3	0	174
6	70.3	18.7	2.3	4.7	0	315
Mean	67.0	13.2	9.4	6.8	0.3	209.2
sem	4.0	2.4	3.9	1.3	0.3	36.6

Table S5: Differential cell counts of IPF BALF.

BAL cell pAkt summary pharmacology

**Donor**		IC50 (nM)	Hill slope
Donor 1		0.345	-1.01
Donor 2		0.655	-1.16
Donor 3		0.673	-1.22
Donor 4		0.432	-1.57
Donor 5		0.982	-1.34
Donor 6		0.613	-1.8
	Geo mean	0.58	
	95% CI	0.40- 0.86	

Table S6: Summary pharmacology of the effect of GSK2126458 on BAL cell pAkt

References

1 Keerthisingam CB, Jenkins RG, Harrison NK*, et al.* Cyclooxygenase-2 deficiency results in a loss of the anti-proliferative response to transforming growth factor-beta in human fibrotic lung fibroblasts and promotes bleomycin-induced pulmonary fibrosis in mice. *Am J Pathol.* 2001; 158: 1411-1422.

2 Crowley C, Klanrit P, Butler CR*, et al.* Surface modification of a POSS-nanocomposite material to enhance cellular integration of a synthetic bioscaffold. *Biomaterials.* 2016; 83: 283-293.

3 Leeming, D. J., D. V. Larsen, C. Zhang, Y. Hi, *et al.* 2010. Enzyme-linked immunosorbent serum assays (ELISAs) for rat and human N-terminal pro-peptide of collagen type I (PINP)--assessment of corresponding epitopes. *Clin.Biochem.* 43:1249-1256.

4 Beal, S., L. B. Sheiner, A. Boeckmann, and R. Bauer 2009. NONMEM User's Guides (1989- 2009). In S. Beal, L. B. Sheiner, A. Boeckmann, and R. Bauer, editors Icon Development Solutions Ellicott City, MD, USA.

5 Chen, C. Z., Y. X. Peng, Z. B. Wang, *et al*. 2009. The Scar-in-a-Jar: studying potential antifibrotic compounds from the epigenetic to extracellular level in a single well. *Br.J.Pharmacol.* 158:1196-1209.
